# Bioaugmentation enhances dark fermentative hydrogen production in cultures exposed to short-term temperature fluctuations

**DOI:** 10.1007/s00253-019-10203-8

**Published:** 2019-11-21

**Authors:** Onyinye Okonkwo, Renaud Escudie, Nicolas Bernet, Rahul Mangayil, Aino-Maija Lakaniemi, Eric Trably

**Affiliations:** 1 LBE, Univ Montpellier, INRA, Narbonne, France; 2 Faculty of Engineering and Natural Sciences, Tampere University, Tampere, Finland

**Keywords:** Biohydrogen, Resilience, Synthetic mixed culture, Bioaugmentation time, Process recovery

## Abstract

Hydrogen-producing mixed cultures were subjected to a 48-h downward or upward temperature fluctuation from 55 to 35 or 75 °C. Hydrogen production was monitored during the fluctuations and for three consecutive batch cultivations at 55 °C to evaluate the impact of temperature fluctuations and bioaugmentation with synthetic mixed culture of known H_2_ producers either during or after the fluctuation. Without augmentation, H_2_ production was significantly reduced during the downward temperature fluctuation and no H_2_ was produced during the upward fluctuation. H_2_ production improved significantly during temperature fluctuation when bioaugmentation was applied to cultures exposed to downward or upward temperatures. However, when bioaugmentation was applied after the fluctuation, i.e., when the cultures were returned to 55 °C, the H_2_ yields obtained were between 1.6 and 5% higher than when bioaugmentation was applied during the fluctuation. Thus, the results indicate the usefulness of bioaugmentation in process recovery, especially if bioaugmentation time is optimised.

## Introduction

Biological methods for H_2_ production, including biophotolysis, photo fermentation, dark fermentation and biocatalysed electrolysis, have received increasing attention due to their ability to utilise renewable feedstocks such as organic wastes, plant biomass residues or sunlight for H_2_ generation (Hallenbeck and Benemann 2002; Dincer 2012). Among the above-stated biological H_2_ production methods, dark fermentation offers several advantages, such as no requirement for light, higher H_2_ production rates, simple bioreactor setup and wide versatility in the choice of the substrate (Chong et al. 2009; Kargi et al. 2012).

Dark fermentative H_2_ production can be carried out at mesophilic, thermophilic and hyperthermophilic conditions (Shin et al. 2004; Kargi et al. 2012) but is thermodynamically more favourable at higher temperatures (Zhang et al. 2014; Zheng et al. 2015). In addition, thermophilic bioprocesses typically result in higher H_2_ production rates as well as inhibition of pathogens (Sahlström 2003) and H_2_ consumers such as methanogens and homoacetogens (Dessì et al. 2018b; Dessì et al. 2018a). However, decreased bacterial diversity at higher temperatures can lead to instability of the bioprocess (Westerholm et al. 2018). Thermophilic processes are typically more sensitive to temperature fluctuations and require more consistent organic loading rate than mesophilic dark fermentation processes (Angelidaki and Ahring 1994). At high loading rates, temperature of anaerobic processes can also increase due to high microbial activity (Daverio et al. 2003; Lindorfer et al. 2006). Possible upsets might also occur much faster at high temperatures due to faster microbial metabolism. However, the extent of the impact of varying bioreactor temperatures depends on the microbial communities present and the magnitude and duration of the temperature change (Okonkwo et al. 2019). A sudden, even transient temperature changes can produce varying responses in microbial populations resulting in an imbalanced metabolism and low process performance (Jiang and Morin 2007; Okonkwo et al. 2019).

In the event of unwanted temperature fluctuation, restoring the activity of the microorganisms catalysing the biological processes can be time-consuming and cost-intensive. One way to stabilise performance of a bioreactor during process disturbances is to augment the bioreactor with H_2_-producing microorganisms (Wang et al. 2008; Ren et al. 2010). Over the years, bioaugmentation has been successfully applied, for example, to reduce the start-up time of dark fermentation (Pandit et al. 2015). Bioaugmentation was shown to protect the existing microbial community from organic loading shocks and reduce the susceptibility of the H_2_-producing bioreactors to process disturbances (Venkata Mohan et al. 2009; Goud et al. 2014; Ács et al. 2015). In a study by Guo et al. (2010), *Escherichia coli*, *Enterobacter aerogenes* and *Bacillus subtilis* were separately used under mesophilic conditions to improve H_2_ production from organic fraction of municipal solid waste (Guo et al. 2010). In another study, Goud et al. (2014) used acidogenic bacteria to enhance the H_2_ production from food waste at an elevated organic load of 50 g L^−1^ of the waste. Bioaugmentation strategy has also been applied as a means to decrease the recovery time of anaerobic digesters stressed by organic overloading (Goud et al. 2014; Acharya et al. 2015).

Different bacterial species possess varied optimal growth requirements and capacities to cope with stress related to fluctuations in cultivation conditions such as changes in temperature or high H_2_ partial pressure (Pawar and van Niel 2013). Therefore, the microorganisms chosen for bioaugmentation purposes should have the desirable properties needed to perform the required function under the specific operational conditions. In our previous study, we showed that effects of temporal temperature fluctuations on dark fermentative H_2_ production were more severe during and after upward temperature fluctuations (from 55 to 65 °C or 75 °C) than during and after downward temperature fluctuations (from 55 to 35 °C and from 55 to 45 °C) (Okonkwo et al. 2019). The aim of this study was to investigate the effects of augmenting thermophilic mixed cultures during and after periods of temperature shock, with a synthetic mixture of well-known H_2_ producers and the importance of bioaugmentation time on dark fermentative H_2_ production. To our knowledge, bioaugmentation with known H_2_-producing bacteria has not been previously studied as a means to resolve the adverse effects caused by sudden temperature fluctuations.

## Materials and methods

### Enrichment culture and medium composition

The inoculum used in this study was obtained from a H_2_-producing thermophilic (55 °C) continuous stirred tank reactor (CSTR), and the medium composition used is as described by Okonkwo et al. (2019). For the enrichment, anaerobically digested sludge heat treated at 90 °C for 20 min was used for inoculation of the CSTR by adding 10% of the sludge to a final working volume of 2 L. The reactor was flushed with nitrogen for 5 min and then operated in continuous mode at hydraulic retention time of 6 h and at 55 °C for a period of 21 days maintaining the pH at 6.5. The enriched microbial community consisted of members belonging to the genus *Thermoanaerobacterium*, *Clostridium* and *Bacillus* (Okonkwo et al. 2019). The medium contained the following compounds in mg L^−1^: K_2_HPO_4_, 500; NH_4_Cl, 100; MgCl_2_·6H_2_O, 120; H_8_FeN_2_O_8_S_2_·6H_2_O, 55.3; ZnCl_2_, 1.0; MnCl_2_·4H_2_O, 2.0; CuSO_4_, 000.4; (NH_4_)_6_Mo_7_O_24_, 1.2; C_O_SO_4_, 1.3; H_3_BO_3_, 0.1; NiCl_2_·6H_2_O, 0.1; Na_2_O_3_Se, 0.01; CaCl_2_·2H_2_O, 80; yeast extract, 500 and 0.055 mL 37% HCl. The culture was fed with glucose (800 mg L^−1^) and xylose (1200 mg L^−1^). Utilising both glucose and xylose is a practical way to move towards efficient bioconversion of lignocellulosic wastes to H_2_.

### Synthetic mixed culture used for bioaugmentation

The following bacterial strains from DSMZ, Germany, were selected for bioaugmentation: *Thermoanaerobacter thermohydrosulfuricus* (DSM-567), *Caldicellulosiruptor saccharolyticus* (DSM-8903), *Clostridium thermocellum* (DSM-1237), *Thermoanaerobacterium thermosaccharolyticum* (DSM-571) and *Thermotoga neapolitana* (DSM-4359). All the species are strictly anaerobic thermophiles except for *T. neapolitana* which can tolerate low oxygen concentration (Van Ooteghem et al. 2004). Together, these bacteria form a synthetic consortium that has the following properties: thermophilic with broad range of temperatures, not auxotrophic to any amino acids and able to degrade wide range of organic substrates (Pawar and van Niel 2013). Table 1 further shows the properties of the bacteria inoculated to the synthetic mixed culture used for bioaugmentation. The bacterial strains were cultivated individually at 65 °C using the medium previously described above, and were then mixed together in a 1:1 ratio based on optical density to obtain the synthetic mixed culture. As individual cultures, the selected microorganisms are efficient H_2_ producers and are able to proliferate in the medium provided for this study. The synthetic culture was cultivated in batch (for 4 days) at 65 °C for three consecutive transfers to a final OD_600_ of 1.1 (on day 4 of the third batch cultivation cycle). The final synthetic culture was then used to study the effect of bioaugmentation during and after the temperature fluctuation periods as described in the next section. Since the bacteria in the consortium have growth temperatures ranging between 55 and 80 °C, 65 °C was considered as suitable temperature for the pre-cultivation. In addition, as our previous study showed that upward temperature fluctuations had more severe impacts on H_2_ production than downward temperature fluctuations (Okonkwo et al. 2019), incubation of the synthetic mixed culture at a temperature higher than 55 °C was hypothesised to provide the needed enhancement of H_2_ production especially in the cultures exposed to upward temperature fluctuations.

**Table 1:** Properties (growth temperature, pH range and oxygen sensitivity) of bacteria inoculated to the synthetic mixed culture used for bioaugmentation

Bacterial species	Temperature (°C)	pH ranges	Oxygen sensitivity	References
*Thermoanaerobacter thermohydrosulfuricus*	55–75	5.8–8.5	Obligate anaerobe	Vos et al. (2011)
*Caldicellulosiruptor saccharolyticus*	65–75	5.2–9	Obligate anaerobe	Vos et al. (2011)
*Clostridium thermocellum*	60–64	5.6–5.8	Obligate anaerobe	Vos et al. (2011)
*Thermoanaerobacter thermosaccharolyticum*	50–80	5.5–9	Obligate anaerobe	Vos et al. (2011)
*Thermotoga neapolitana*	55–95	5.5–9	Obligate anaerobe but tolerates low oxygen	Jannasch et al. (1988); Van Ooteghem et al. (2004)

### Experimental procedure

The batch experiments were carried out in gas-tight 500-mL polypropylene centrifuge tubes (69 × 160 mm, Beckman Coulter) designed for gas and liquid sampling as well as centrifugation and cultivation. The microbial inoculum used in each batch corresponded to 10% of the total working volume (200 mL). Each cultivation tube was flushed with nitrogen for 5 min before starting the incubation.

**Fig. 1:**
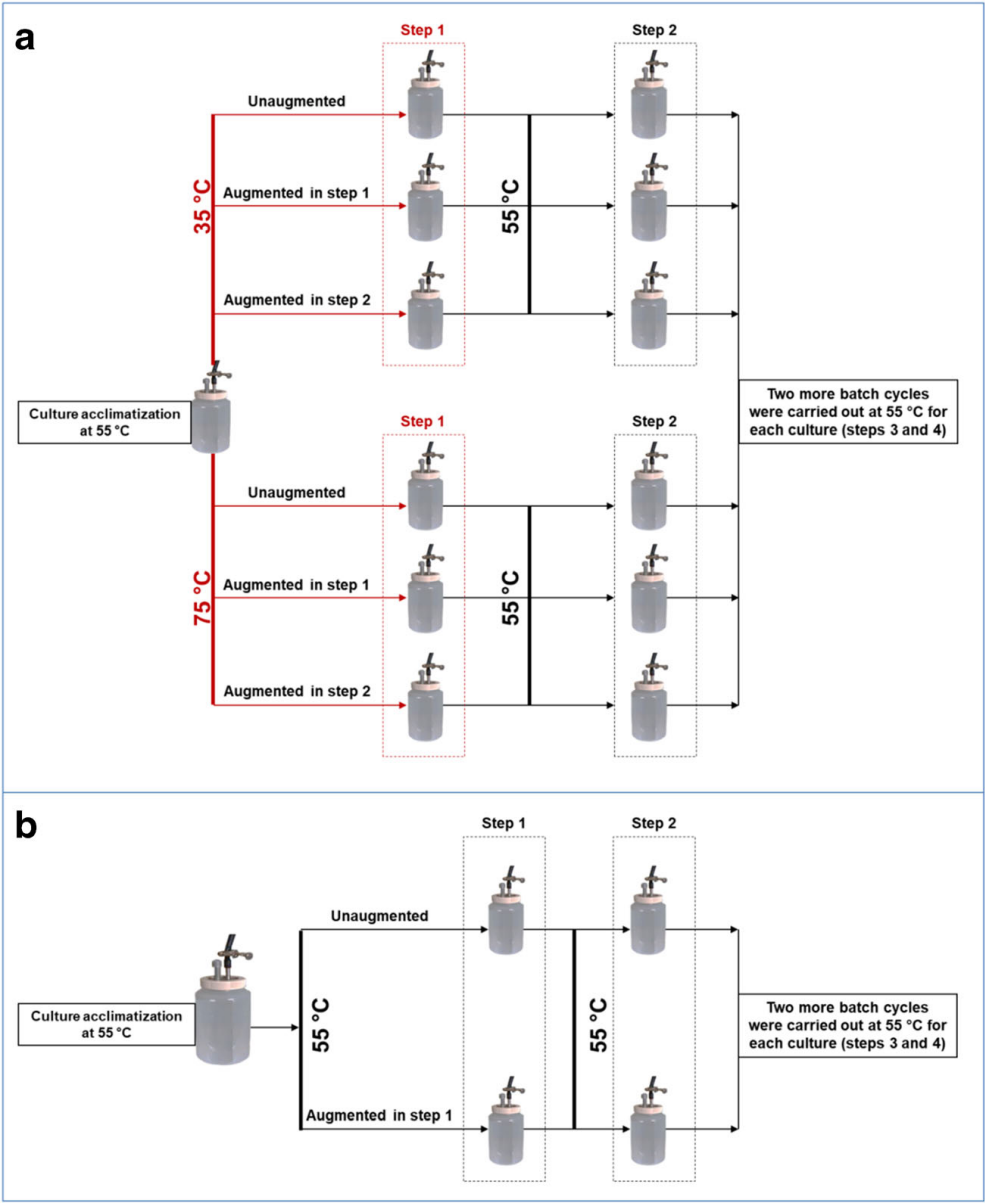
Experimental set-up to study the effects of bioaugmentation with a synthetic mixed culture on H_2_ production during and after temperature stress periods. All cultures were first incubated in batch at 55 °C for 48 h. Then, some of the cultures were subjected to a 48-h temperature fluctuation at 35 or 75 °C (step 1) and subsequently incubated at 55 °C for three consecutive 48-h batch cultivation steps (steps 2, 3 and 4) (**a**). The experiment included also control cultures, which were incubated after the acclimatization period at 55 °C for four consecutive batch cultivation steps with and without bioaugmentation in step 1 (**b**)

Prior to exposing the cultures to temporal temperature fluctuations, the mixed culture inoculum was first acclimatised to batch growth conditions by incubating at 55 °C for 48 h as shown in Fig. 1. At 55 °C, the substrates were completely consumed by the end of the 48-h period. After the acclimatisation, the cultures were subjected to either downward (from 55 to 35 °C) or upward temperature fluctuation (from 55 to 75 °C) of 48 h (referred to as step 1 in Fig. 1) to evaluate the impact of temperature fluctuation on H_2_ production. After the stress period, each culture was centrifuged for 5 min; the spent medium was decanted and replaced with fresh medium, and the culture was then incubated again at 55 °C for 48 h. This was carried out for altogether three consecutive batch cultivations (steps 2, 3 and 4) to estimate the H_2_ production recovery after the temperature fluctuations. To determine the impact of bioaugmentation, cultures exposed to similar conditions (downward or upward temperature fluctuation) were augmented with the synthetic mixed cultures described in the previous section, either during or after the temperature fluctuation period (in the beginning of step 1 or in the beginning of step 2). The reason for the different bioaugmentation times was to monitor the effect of bioaugmentation time on H_2_ production. As a control, batch cultivations were also carried out at 55 °C for four consecutive cycles (steps 1 to 4). The cultures were either unaugmented or augmented with the synthetic mixed culture in the beginning of step 1, to study the effect of bioaugmentation under stable incubation temperature. The synthetic mixed culture/inoculum (volume of synthetic mixed culture to volume of mixed culture inoculum) ratio that was used for enhancing H_2_ production during or after temperature fluctuation was 0.2. This bioaugmentation ratio was chosen, because according to Sharma and Melkania (2018), the highest cumulative H_2_ production and volumetric H_2_ production was obtained with the bacteria/sludge ratio of 0.2 and 0.25, when they studied the effect of bioaugmentation on H_2_ production from organic fraction of municipal solid waste.

The pH during the experiments was adjusted to 6.5, and the incubation temperatures were attained using temperature-controlled water baths. The experimental design described is similar to that employed by Okonkwo et al. (2019) to study the impact of temperature fluctuations on dark fermentative H_2_ production.

### Analytical techniques and calculations conducted

Glucose, xylose, organic acid and alcohol concentrations were measured by high-performance liquid chromatography (HPLC) using a refractive index detector (Waters R410) as described previously by Monlau et al. (2013). The gas composition was analysed by a gas chromatograph (Clarus580, Perkin Elmer, Waltham, USA) equipped with a thermal conductivity detector (399152, Linde, Munich, Germany) and two columns: RtQBond to split H_2_, O_2_, N_2_ and CH_4_ and RtMolsieve (5Å) to quantify CO_2_. The carrier gas was argon at a pressure of 3.5 bar. The temperatures of the oven, injector and detector were 60 °C, 250 °C and 150 °C, respectively. The gas volume and composition measurements were conducted at the respective incubation temperatures mentioned in “Experimental procedure” by retaining the cultivation bottles in the water baths during the sampling. The total volume of produced H_2_ was calculated at standard temperature using Eq. 1 (Logan et al. 2002).

1 $$ {V}_{{\mathrm{H}}_2,t}={V}_{{\mathrm{H}}_2,t-1}+{C}_{{\mathrm{H}}_2,t}\left({V}_{G,t}-{V}_{G,t-1}\right)+{V}_H\left({C}_{{\mathrm{H}}_2,t}-{C}_{{\mathrm{H}}_2,t-1}\right) $$

where ${\mathrm{V}}_{{\mathrm{H}}_2,t}$ is the cumulative H_2_ gas produced at time t, ${V}_{{\mathrm{H}}_2,t-1}$ is the cumulative H_2_ gas produced at *t* − 1, *V*_*G*, *t*_ is the total gas volume at time *t*, *V*_*G*, *t* − 1_ is the total gas volume at time *t* − 1, ${C}_{{\mathrm{H}}_2,t}$ is the H_2_ gas fraction in the headspace at time *t*, ${C}_{{\mathrm{H}}_2,t-1}$ is the H_2_ gas fraction in the headspace at time *t* − 1 and *V*_*H*_ is the total headspace volume in the culture bottle. H_2_ production in moles was calculated on the basis that 1 mol of an ideal gas will occupy a volume of 22.4 L at standard temperature and pressure according to the ideal gas law. H_2_ yield was calculated by dividing the mol H_2_ per mole of hexose equivalent using the conversion factor of 5/6 for converting xylose to its hexose equivalent. Total chemical oxygen demand (COD) of soluble compounds was calculated based on the sum of acids, ethanol and residual sugars by using the following conversion factors: 1 mM glucose = 192 mg COD L^−1^, 1 mM xylose = 160 mg COD L^−1^, 1 mM acetate = 64 mg COD L^−1^, 1 mM propionate = 112 mg COD L^−1^, 1 mM lactate = 96 mg COD L^−1^, 1 mM butyrate = 160 mg COD L^−1^ and 1 mM ethanol = 96 mg COD L^−1^ (Sivagurunathan and Lin 2016; Gonzales and Kim 2017). The relative H_2_ yield obtained during and after the temperature fluctuations was calculated using the results obtained in the unaugmented control using Eq. 2:

2 $$ \mathrm{Relative}\ {\mathrm{H}}_2\ \mathrm{yield}\ \left(\%\right)=\frac{{\mathrm{H}}_2\ \mathrm{yield}\ \mathrm{obtained}\ \mathrm{during}/\mathrm{after}\ \mathrm{temperature}\ \mathrm{shift}\ }{average\ {\mathrm{H}}_2\  yield\ obtained\ from\ the\ unaugmented\ control}\times 100 $$

### Microbial analysis for unaugmented and augmented samples during the temperature fluctuation

Genomic DNA was extracted using the PowerSoil DNA Isolation Kit (MoBio Laboratories, Inc., Carlsbad, CA, USA) according to the manufacturer’s instructions. Primers 515_532U and 909_928U (Wang and Qian 2009) including their respective linkers were used to amplify the V4_V5 region of the 16S rRNA gene. The resulting products were purified and sequenced using Illumina MiSeq. Sequencing and library preparation were performed at the Genotoul Lifescience Network Genome and Transcriptome Core Facility in Toulouse, France. Sequence analysis was performed as previously described by Venkiteshwaran et al. (2016). The 16S rRNA sequences used to support the findings of this study have been deposited in the NCBI Sequence Read Archive under project file SUB6057113: MN203768–MN203978.

## Results

### Characterisation of bacteria in the synthetic mixed culture

**Fig. 2:**
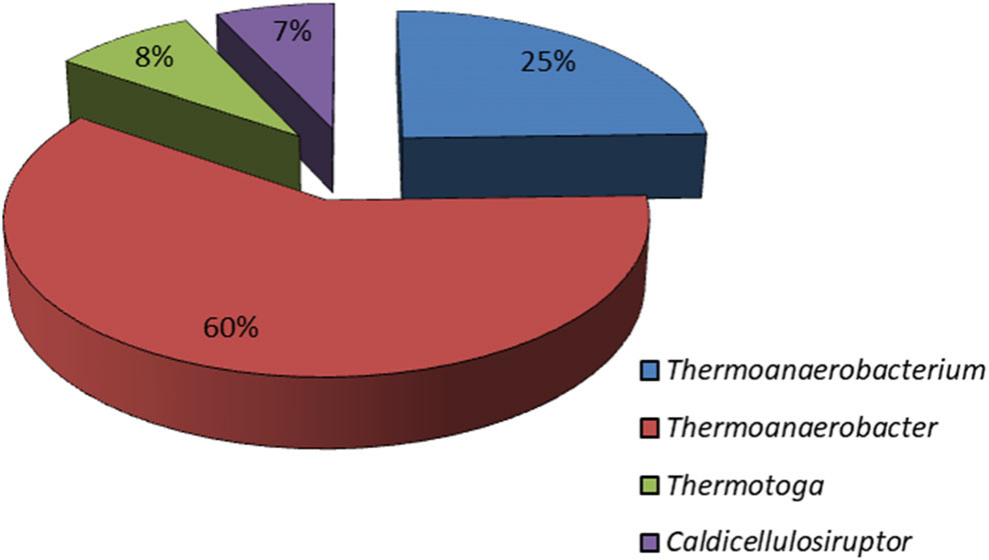
Relative abundance (%) of each genera in the synthetic mixed culture used for bioaugmentation of the native mixed culture during and after the temperature fluctuations

Bacteria belonging to the genera *Thermoanaerobacter*, *Caldicellulosiruptor*, *Clostridium*, *Thermoanaerobacterium* and *Thermotoga* were added to the synthetic mixed culture that was used for bioaugmentation in this study. After incubation of the synthetic mixed culture for three consecutive 4-day batch cultivations at 65 °C, the microbial characterisation revealed that the community included all the added bacterial genera with the exception of *Clostridium* (Fig. 2). Compared with all the other bacteria in the culture, *Thermoanaerobacter* was seen to have the highest relative abundance (60%), followed by *Thermoanaerobactium* (25%)*. Thermotoga* and *Caldicellulosiruptor* had an abundance of 8 and 7%, respectively.

### Comparison between augmented and unaugmented cultures at constant temperature of 55 °C

All the substrates (800 mg L^−1^ glucose and 1200 mg L^−1^ xylose) added to the unaugmented control cultures incubated at constant temperature of 55 °C were consumed within the 48-h period. The H_2_ yield obtained from the unaugmented cultures was 1.85 ± 0.01, 1.80 ± 0.03, 1.86 ± 0.06 and 1.89 ± 0.10 mol H_2_ mol^−1^ hexose equivalent in steps 1, 2, 3 and 4, respectively, resulting in an average H_2_ yield of 1.85 ± 0.04 mol H_2_ mol^−1^ hexose equivalent.

To determine the influence of bioaugmentation without any temperature stress, cultures incubated at constant temperature of 55 °C were augmented with the synthetic mixed culture in the beginning of step 1. The H_2_ yield obtained in the bioaugmented control cultures was 2.19 ± 0.08, 2.07 ± 0.06, 2.07 ± 0.16 and 1.94 ± 0.01 mol H_2_ mol^−1^ hexose equivalent in steps 1, 2, 3 and 4, respectively. Thus, the average H_2_ yield was 2.07 ± 0.09 mol H_2_ mol^−1^ hexose equivalent. The average H_2_ yield obtained with bioaugmentation was significantly higher than the average yield obtained from the unaugmented control cultures. A one-way ANOVA between the unaugmented and the augmented control cultures showed that the difference in H_2_ yield was statistically significant (*p* < 0.05).

**Fig. 3.**
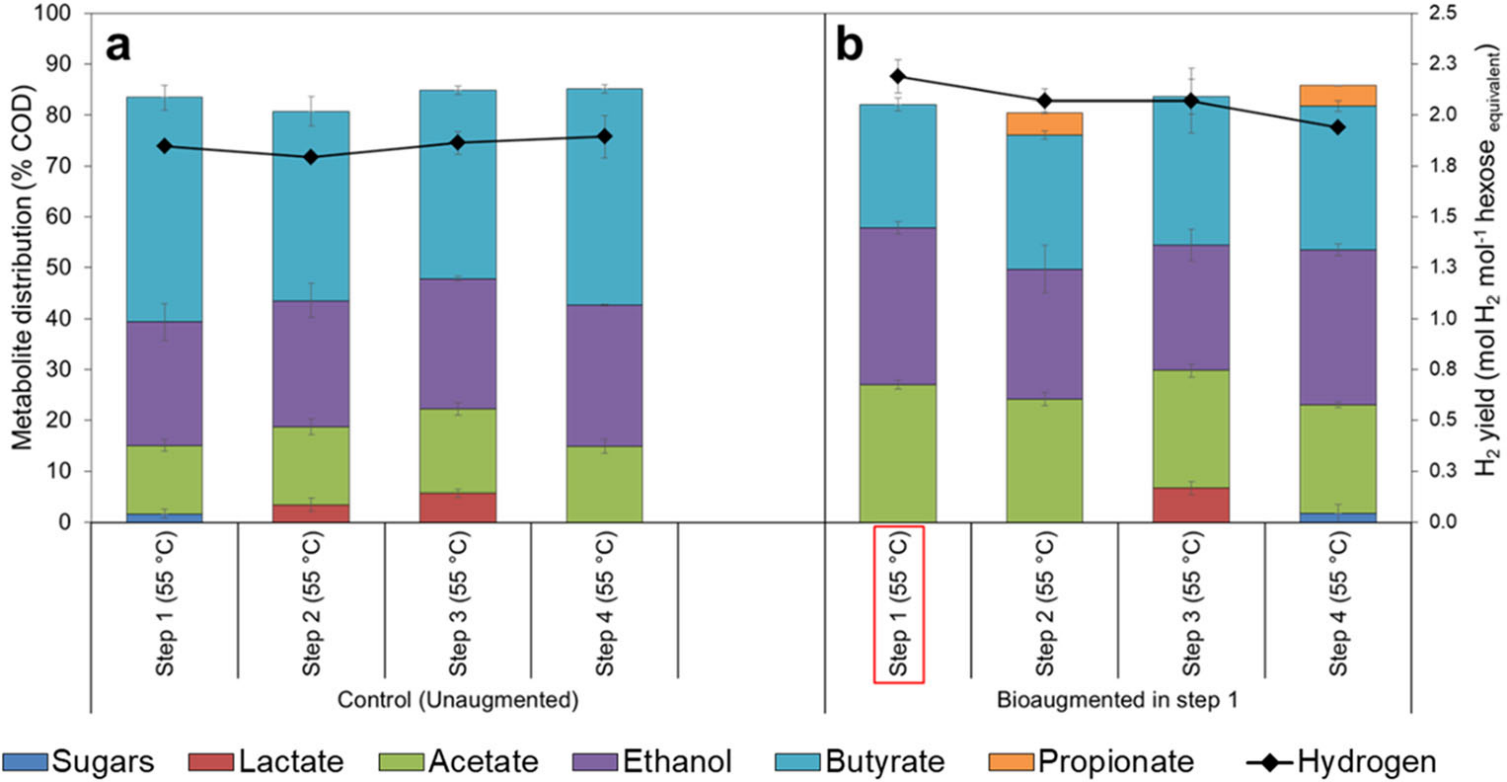
Hydrogen yield and the contribution of the residual sugars and soluble metabolites to the endpoint COD at 55 °C in **a** the unaugmented control cultures and **b** in the augmented control cultures. Data represents mean values and standard deviation from duplicate cultivations. The red rectangle indicates the point at which bioaugmentation was applied

Bioaugmentation had also a clear impact on the distribution of the soluble metabolites. The metabolites observed in all conditions included acetate, butyrate, ethanol and traces of lactate and propionate. The unaugmented cultures had a higher percentage of butyrate in all steps (Fig. 3a), while bioaugmentation altered the metabolic profile by increasing the share of acetate production (Fig. 3b). Propionate was also detected in steps 2 and 4 in the bioaugmented cultures.

To further evaluate whether the increase in H_2_ production and metabolite shift was directly a result of the bioaugmentation, the microbial communities in the unaugmented and augmented cultures incubated at constant temperature of 55 °C were evaluated using microbial samples taken in the end of incubation step 1. Based on the results obtained, the unaugmented control culture composed of *Thermoanaerobacterium* (73%), *Clostridium* (2%), *Bacillus* (10%) and *Desulfitobacterium* (3%). Other genera represented about 11% of the total relative abundance. The microorganisms found in the bioaugmented cultures included *Thermoanaerobacterium* (85%), *Clostridium* (1%), *Bacillus* (4%) and others (10%).

### Process recovery after the downward temperature shift and the impact of bioaugmentation

**Fig. 4:**
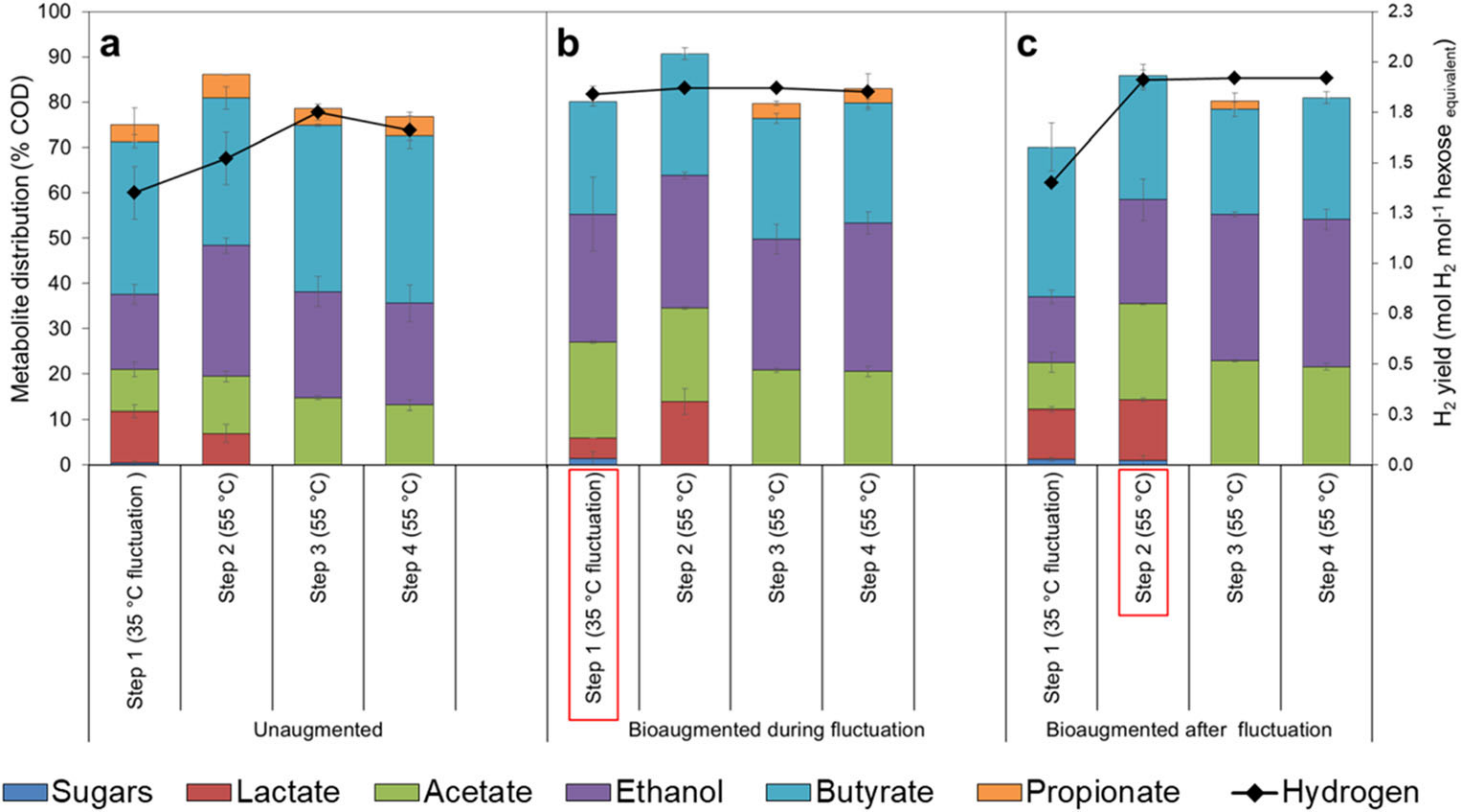
Hydrogen yield and the contribution of the residual sugars and soluble metabolites to the endpoint COD during and after the downward temperature fluctuation from 55 to 35 °C **a** without bioaugmentation, **b** with bioaugmentation applied in the beginning the temperature fluctuation (step 1) and **c** with bioaugmentation applied after the temperature fluctuation in the beginning of step 2. Data represents mean values and standard deviation from duplicate cultivations. The red rectangles indicate the point at which bioaugmentation was applied

A downward temperature fluctuation during dark fermentation was shown to have a negative impact on H_2_ production in the unaugmented cultures. The temperature shift from 55 to 35 °C in step 1 resulted in a H_2_ yield of 1.35 ± 0.13 mol H_2_ mol^−1^ hexose equivalent (Fig. 4a), which is 27% lower than the average H_2_ yield obtained under stable conditions with the unaugmented control (Fig. 3a). After the temperature fluctuation period, the H_2_ yield increased gradually from step 2 to step 3 (Fig. 4a). The H_2_ yields obtained in steps 2, 3 and 4 (after temperature fluctuation) were 1.52 ± 0.13, 1.75 ± 0.04 and 1.66 ± 0.09 mol H_2_ mol^−1^ hexose equivalent, respectively.

Cultures to which the bioaugmentation was applied at the beginning of temperature fluctuation (step 1) gave a H_2_ yield of 1.84 ± 0.04 mol H_2_ mol^−1^ hexose equivalent during the fluctuation. The obtained H_2_ yield was similar to that observed with unaugmented control cultures incubated at 55 °C (1.85 ± 0.04 mol H_2_ mol^−1^ hexose equivalent), which indicated that the negative impact caused by the downward temperature fluctuation could be compensated by bioaugmentation with the synthetic mixed culture. The H_2_ yield further increased to a maximum of 1.87 ± 0.01 mol H_2_ mol^−1^ hexose equivalent in step 3 when the temperature was returned to 55 °C (Fig. 4b).

In cultures to which the bioaugmentation was applied after the temperature fluctuation (step 2), H_2_ yield was higher than in the cultures to which the bioaugmentation was applied at the beginning of the temperature fluctuation, being 1.91 ± 0.05, 1.92 ± 0.02 and 1.92 ± 0.01 mol H_2_ mol^−1^ hexose equivalent in steps 2, 3 and 4, respectively (Fig. 4c).

The total soluble metabolic end-products calculated based on their COD were between 74 and 90% of the initial COD added as xylose and glucose (Fig. 4). In the unaugmented cultures, the metabolites produced during the temperature fluctuation (step 1) were acetate, butyrate, ethanol, lactate and propionate (Fig. 4a). The shift in temperature back to 55 °C caused a shift in the distribution of the soluble metabolic products as acetate and ethanol showed a slight increase while the concentration of lactate reduced significantly and was not at all detected in steps 3 or 4 (Fig. 4a). In cultures to which the bioaugmentation was applied during the temperature fluctuation, the share of acetate and ethanol increased significantly compared with the unaugmented cultures. Lactate slightly increased from step 1 to step 2 but was not detected in steps 3 and 4. Meanwhile, very low concentrations of propionate were also detected in steps 3 and 4 (Fig. 4b). When the bioaugmentation was applied after the temperature fluctuation, acetate and ethanol share also increased compared with the unaugmented culture. Lactate was detected in step 2 but was not detected in steps 3 and 4 (Fig. 4c), similarly to the cultures to which bioaugmentation was conducted during the fluctuation (Fig. 4b).

During the downward temperature shift, *Thermoanaerobacterium* spp. was less dominant in the unaugmented cultures compared with incubation at 55 °C with a share of 27%, while *Clostridium* and *Bacillus* had a relative abundance of 22% and 31%, respectively. Other genera accounted for 18% of the community. Bioaugmentation in the beginning of the temperature fluctuation caused an increase in the relative abundance of *Thermoanaerobacterium* spp. from 27% in the unaugmented culture to 72%, which suggests that the *Thermoanaerobacterium* added was involved in H_2_ production during the downward temperature fluctuation despite the low temperature.

### Process recovery after the upward temperature shift and impact of bioaugmentation

**Fig. 5:**
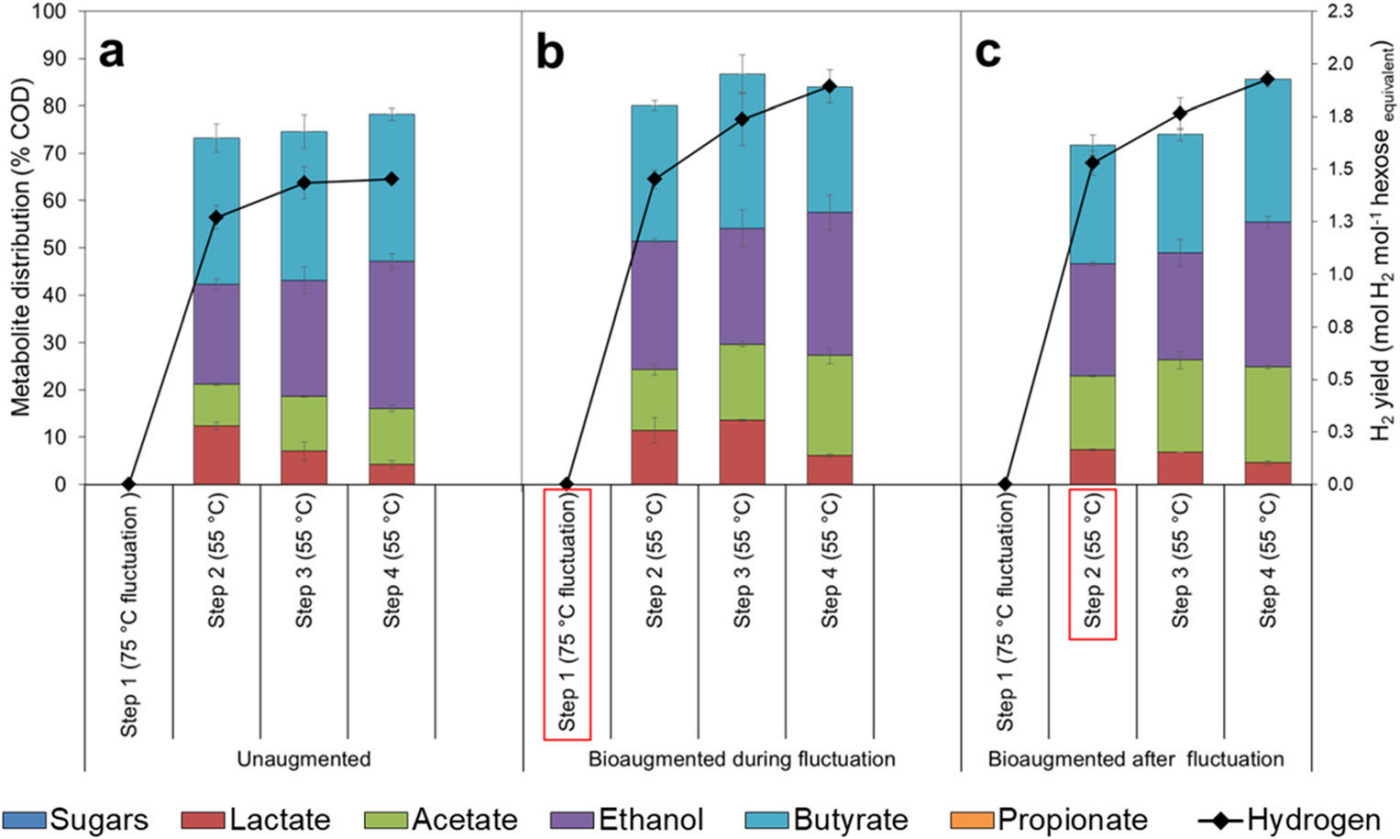
H_2_ yield and the contribution of the residual sugars and soluble metabolites to the endpoint COD during and after the downward temperature fluctuation from 55 to 75 °C **a** without bioaugmentation, **b** with bioaugmentation applied during temperature fluctuation (step 1) and **c** with bioaugmentation applied after temperature fluctuation in the beginning of step 2. Data represents mean values and standard deviation from duplicate cultivations. The red rectangles indicate the point at which the bioaugmentation was applied

Upward temperature fluctuation from 55 to 75 °C was shown to have a severe impact on microbial metabolism as no H_2_ production was observed within the 48-h incubation period in the unaugmented culture or in the cultures to which bioaugmentation was done in the beginning of the temperature fluctuation (step 1). In the unaugmented cultures, when the temperature was taken back to 55 °C (step 2), H_2_ production occurred (1.27 ± 0.06 mol H_2_ mol^−1^ hexose equivalent) and gradually increased to 1.43 ± 0.08 and 1.45 ± 0.01 mol H_2_ mol^−1^ hexose equivalent in steps 3 and 4, respectively (Fig. 5a). However, the highest H_2_ yield which was obtained in step 4 was still significantly lower (approximately 21%) than the average H_2_ yield obtained under stable temperature conditions (unaugmented control at 55 °C).

In the cultures augmented in the beginning of the temperature fluctuation period, H_2_ yield was 1.45 ± 0.05, 1.74 ± 0.13 and 1.89 ± 0.08 mol H_2_ mol^−1^ hexose equivalent in steps 2, 3 and 4, respectively (Fig. 5b). The H_2_ production was higher than in the unaugmented culture exposed to upward temperature fluctuation, and the highest H_2_ yield obtained (in step 4) was comparable with the H_2_ yield in the unaugmented control kept at 55 °C. In the cultures augmented after the temperature fluctuation period (in step 2), H_2_ yield was 1.53 ± 0.06, 1.76 ± 0.08 and 1.93 ± 0.04 mol H_2_ mol^−1^ hexose equivalent in steps 2, 3 and 4, respectively (Fig. 5c).

During the upward temperature fluctuation, no VFAs and alcohols were formed in the unaugmented or in the cultures augmented in the beginning of the fluctuation, which further verified the absence of microbial activity. In the unaugmented cultures, the metabolites produced were acetate, butyrate, ethanol and lactate, when the temperature was shifted back to 55 °C (Fig. 5a). Butyrate had the highest share of produced soluble metabolites, followed by ethanol and acetate. Lactate, which contributed 12% in step 2, reduced to 4% in step 4, while the share of acetate and ethanol increased slightly. However, the contribution of butyrate remained relatively constant (Fig. 5a). In the cultures to which the bioaugmentation was applied in the beginning of the temperature fluctuation, butyrate remained the most abundant soluble metabolite (Fig. 5b). In the cultures to which the bioaugmentation was applied after the temperature fluctuation, there was also increased acetate and ethanol production observed and the share of lactate reduced gradually with each incubation step (Fig. 5c), similar to the cultures augmented in the beginning of the fluctuation (Fig. 5b).

## Discussion

In this study, it was observed that bioaugmenting native microbial communities with synthetic mixed cultures during and after upward or downward temperature fluctuation enhanced H_2_ production and thus limited the negative impact observed in the control cultures. Prior to the augmentation, microbial data of the synthetic mixed culture used showed differences in the microbial distribution with *Thermoanaerobacter* having a higher relative abundance (60%) than the other species added, followed by *Thermoanaerobacterium*, *Thermotoga* and *Caldicellulosiruptor* (Fig. 2). The difference in the relative distribution observed in the synthetic mixed culture was likely a result of the different growth rates of the different bacteria at the selected growth conditions (Vanfossen et al. 2009; Yu and Drapcho 2011; Akinosho et al. 2014). Of all the species added to the synthetic culture, only *C. thermocellum* was not detected in the final synthetic mixed culture used for bioaugmenting. This was likely because the ability of the other bacteria to utilise xylose gave them a competitive advantage over *C. thermocellum*, since it does not metabolise xylose (Wilson et al. 2013) and has been shown to grow poorly on glucose (Ng and Zeikus 1982). The preferred soluble sugars of *C. thermocellulum* are cellulose, cellobiose or cellodextrins (Stevenson and Weimer 2005; Zhang and Lynd 2005). Therefore, *C. thermocellulum* was already lost before the bioaugmentation of the synthetic cultures (Fig. 2).

Compared with the unaugmented control culture incubated at constant temperature of 55 °C, the bioaugmented control cultures demonstrated an increase in H_2_ production compared with the unaugmented cultures. Additionally, the relative abundance of *Thermoanaerobacterium* spp. increased in the augmented cultures compared with the unaugmented cultures. Relating this observation to H_2_ production suggests that *Thermoanaerobacterium* spp. had the most significant impact on the H_2_ production and might have influenced the increase in acetate concentration since it is capable of producing large amounts of acetate (O-Thong et al. 2008; Cao et al. 2010). The relative abundance of the other genera representing the added species was quite low (*Caldicellulosiruptor* had a relative abundance of 0.02%, *Thermoanaerobacter*, 0.3% and *Thermotoga*, 0.04%).

For the cultures exposed to the downward temperature fluctuation, the relative H_2_ yield (H_2_ yield compared with the unaugmented control) after the temperature fluctuation period was 5 to 10% lower than the H_2_ yield obtained the unaugmented control at 55 °C. This implied that the downward temperature fluctuation caused a reduction in H_2_ yield even after three consecutive batch incubations at 55 °C. Gadow et al. (2013) reported similar observations when a H_2_-producing stirred tank reactor operated in a continuous mode with hydraulic retention time of 10 days was exposed to 24-h temperature fluctuation from 52 to 32 °C. They demonstrated a decrease of 27% in the H_2_ content during the temperature shock. Furthermore, the maximum H_2_ content they achieved after 10 days of recovery (at 55 °C) was 9% lower than the value before the temperature fluctuation. As shown in Fig. 4c, H_2_ production was enhanced when bioaugmentation was applied in the beginning of the temperature fluctuation (in step 1). However, based on the differences in H_2_ production observed with the different bioaugmentation times, the short-term temperature fluctuation also caused stress on the microorganisms used in the bioaugmentation even though the differences were not statistically significant (*p* > 0.05). Thus, in order to maximise the recovery of H_2_ production after downward temperature fluctuations, it seems advisable to conduct the bioaugmentation after the fluctuation period. Alternatively, repeated bioaugmentation applied as soon as an unwanted temperature fluctuation is observed and after the temperature has been restored to a desired level might also enable maximal process recovery after a temperature fluctuation period. For example, Yang et al. (2016a, b) showed improved performance of anaerobic digestion with the application of repeated bioaugmentation.

The addition of synthetic mixed cultures during or after downward temperature fluctuation showed a positive impact in the enhancement of H_2_ production and improved the recovery time of bacterial activity when compared with the unaugmented cultures. Comparison between the unaugmented and the augmented cultures exposed to the downward temperature fluctuation showed that *Thermoanaerobacterium* spp. added into the microbial consortium played a significant role in the H_2_ production observed during the downward temperature fluctuation. Other species present in the synthetic mixed culture used for bioaugmentation had a much lower relative abundance during the downward temperature fluctuation, as *Thermoanaerobacter* had a relative abundance of 0.8%, *Caldicellulosiruptor* 0.02% and *Thermotoga* 0.2%. Thus, they might have had little or no influence on the enhancement of H_2_ production, although Rafrafi et al. (2013) have reported that, despite low levels of abundance, subdominant species are able to influence the global microbial metabolic network in mixed cultures.

Cultures exposed to the upward temperature fluctuation had a completely different response to H_2_ production when compared with the cultures exposed to the downward temperature fluctuation. During the upward temperature fluctuation (75 °C), no metabolic activity was observed for the 48-h period. However, this did not imply complete deterioration of the culture, as microbial activity was observed when the temperature was brought back to the original incubation temperature of 55 °C. Nonetheless, H_2_ production observed was significantly lower compared with the control cultures maintained at 55 °C. It was unexpected that no H_2_ production was observed in the cultures augmented in the beginning of the upward temperature fluctuation as the synthetic mixed culture used for the bioaugmentation contained *Caldicellulosiruptor* and *Thermotoga* capable of producing H_2_ at extremely high temperatures of 70 and 80 °C, respectively (Abreu et al. 2016). Additionally, based on the wide temperature and pH range of *T. neapolitana*, it was expected that it would have been an excellent member of the microbial community during the temperature fluctuation at 75 °C. Nonetheless, it is possible that the 48-h fluctuation period was too short for the bacteria to get adapted to the high temperature, which is why there was no sign of microbial activity observed. When comparing the cultures which were augmented at different times, higher H_2_ yields were obtained when the bioaugmentation was applied after the upward temperature fluctuation than when the augmentation was applied in the beginning of the temperature fluctuation. Although the differences in H_2_ yield obtained from the different bioaugmentation times were not statistically significant (*p* > 0.05), it is likely that also the bacteria used for bioaugmentation were negatively affected by the upward temperature stress. Nonetheless, bioaugmentation proved to be an effective strategy for enhancing H_2_ production after the temperature stress. Furthermore, the bioaugmentation is also considered important for boosting the microbial diversity especially after upward temperature fluctuations, as even short-term upward temperature fluctuations have been demonstrated to result in loss of microbial diversity (Gadow et al. 2013; Okonkwo et al. 2019).

It was expected that *Thermoanaerobacter thermohydrosulfuricus* would be an active participant in the consortium during the downward or upward temperature fluctuation due to its relatively high abundance (60%) observed in the synthetic mixed culture (Fig. 2). Furthermore, *Thermoanaerobacter* has been shown to grow in conditions, which are similar to the cultivation conditions used in this study (Table 1). Even though *Thermoanaerobacter* was the dominant genus in the synthetic mixed cultures prior to augmentation, while the results obtained from the augmented cultures showed that *Thermoanaerobacterium* became the most dominant species during or after bioaugmentation under all studied conditions except in unaugmented cultures undergoing downward temperature fluctuation. It is possible that the pre-existence and dominance of *Thermoanaerobacterium* spp. prior to augmentation ensured optimal growth/survival of the specie and better metabolic adaptation compared with the other species added in the consortium. It has been reported that *Thermoanaerobacterium thermosaccharolyticum* (which was among of the bacteria in the synthetic mixed culture) is able to grow at 35–37 °C if spores are first germinated at a higher temperature (Ashton 1981). Hence, the dominance of *Thermoanaerobacterium* spp. might have been as a result of its ability to better cope with temperature stress as opposed to the other species. As seen in Figs. 4 and 5 between unaugmented and augmented cultures, stress factors such as temperature slow adaptation time and might have prevented the activity of the other species or their proliferation. Chen et al. (2015) demonstrated that the successful application of bioaugmentation relied upon the adaptation or coexistence of the bioaugmented bacteria to indigenous microorganisms. The increase in the abundance of *Thermoanaerobacterium* in the augmented cultures caused a relative decrease in abundance of the other microbial genera in the consortium.

The addition of bacteria into a native consortium has been shown in previous studies to affect the metabolic distribution, and depending on the metabolic pathways utilised by the bacteria added, an additional pathway might be observed (Yang et al. 2016a). It is therefore important to choose bacteria, which are directly involved with H_2_ production, for bioaugmentation. Furthermore, it is likely that during the heat shock, some of the microorganisms formed spores as a mechanism to overcome the heat shock, which would explain the gradual increase in H_2_ yield from steps 2 to 4 after the upward temperature fluctuation. For example, *Thermoanaerobacterium* has been reported to form spores, which are heat resistant (Lee et al. 1993; Mtimet et al. 2016). Thus, the strengthening of a microbial consortium by bioaugmentation improves recovered activity during or after stress periods. This study demonstrated that bioaugmenting a H_2_-producing mixed culture with a synthetic mixed culture consisting of known H_2_-producing bacteria can be used as an effective approach for enhancing H_2_ production performance during temperature fluctuations. However, the positive effects of bioaugmentation were even higher, when it was applied after the temperature fluctuation. Thus, bioaugmentation both during and after the temperature fluctuation could also be a valid option.
